# Burden and Impact of Acute Gastroenteritis and Foodborne Pathogens in Trinidad and Tobago

**Published:** 2013-12

**Authors:** Carelene Lakhan, Neela Badrie, Adash Ramsubhag, Kumar Sundaraneedi, Lisa Indar

**Affiliations:** ^1^Faculty of Food and Agriculture, University of the West Indies; ^2^Faculty of Science and Technology, University of the West Indies; ^3^Ministry of Health, Trinidad and Tobago; ^4^Caribbean Epidemiology Centre (CAREC/PAHO/WHO), Trinidad and Tobago

**Keywords:** Burden of acute gastroenteritis, Diarrhoea, Foodborne disease, Foodborne illness, Population survey, Surveillance systems, Trinidad and Tobago

## Abstract

Objectives of this study were to determine the burden and impact of acute gastroenteritis (AGE) and foodborne pathogens in Trinidad and Tobago. A retrospective, cross-sectional population survey, based on self-reported cases of AGE, was conducted in November-December 2008 and May-June 2009 (high- and low-AGE season respectively) by face-to-face interviews. From 2,145 households selected to be interviewed, the response rate was 99.9%. Of those interviewed, 5.1% (n=110; 95% CI 4.3-6.2) reported having AGE (3 or more loose watery stools in 24 hours) in the 28 days prior to the interview (0.67 episodes/person-year). Monthly prevalence of AGE was the highest among children aged <5 years (1.3 episodes/year). Eighteen (16%) persons with AGE sought medical care (4 treated with oral rehydration salts and 6 with antibiotics), and 66% reported restricted activity [range 1-16 day(s)]. The mean duration of diarrhoea was 2.3 days (range 2-10 days). One case submitted a stool sample, and another was hospitalized. Overall, 56 (10%) AGE specimens tested positive for foodborne pathogens. It was estimated that 135,820 AGE cases occurred in 2009 (84% underreporting), and for every 1 AGE case reported, an additional 6.17 cases occurred in the community. The estimated economic cost of AGE ranged from US$ 27,331 to 19,736,344. Acute gastroenteritis, thus, poses a huge health and economic burden on Trinidad and Tobago.

## INTRODUCTION

The World Health Organization (WHO) noted that all cases of gastroenteritis are not foodborne, and all foodborne diseases do not cause gastroenteritis. However, food does represent an important vehicle for pathogens of great public-health attention. Acute gastroenteritis (AGE) and diarrhoea are clinical outcomes of foodborne diseases (FBDs), with data showing that up to 70% of AGE results from FBD (1). AGE is a major cause of morbidity and mortality worldwide and, therefore, is an important global public-health issue in both developed and developing countries (2-7).

Trinidad and Tobago (T&T) is a Twin-island State situated at the southern end of the Caribbean, with mainly an energy-driven economy (8). Having gained its independence in August 1962, the country is a democratic republic within the British Commonwealth. Trinidad is currently organized into 13 administrative areas or Regional Corporations as set up under the 1981 Regional Corporation Act. The 2010 Census data showed a population of 1,226,383 (621,631 males: 604,752 females) (9). In 1994, the Regional Health Authorities Act was enacted, establishing five Regional Health Authorities (RHAs)—four in Trinidad and one in Tobago—as independent statutory authorities accountable to the Minister of Health. The RHA territories have been drawn to coincide with those of local governments (the Regional Corporations) to ensure that they effectively coordinate among the latter in providing a range of health services to their catchment populations.

Communicable diseases are an important cause of morbidity and death in T&T, causing 42% of total illness and 7% of deaths. They are the second-most frequent cause of admission to hospitals at acute stage (10). Little information is known on the burden and aetiology of AGE in T&T. During 2000-2005, there were seven large outbreaks of AGE with over 20,000 cases reported per year but less than 70 cases were of known aetiology (11). The national surveillance system for AGE in T&T is based on both syndromic cases of AGE and its laboratory-confirmed pathogens collected using standard data-collection forms—weekly syndromic and monthly laboratory data-collection forms (11)—based on the Caribbean Public Health Agency (CARPHA), formerly known as the Caribbean Epidemiology Centre (CAREC). Syndromic AGE surveillance collects data on weekly basis on the number of persons presenting with AGE symptoms at local healthcare facilities. Laboratory-based foodborne pathogens and AGE surveillance collects monthly data from the major hospitals and laboratories that process and test AGE-related stool specimens to identify the number of AGE cases whose stool samples test positive for a related pathogen (12).

The reason why these illnesses are not well-understood lies in the fact that most affected people are not captured by the National Surveillance Unit (NSU). The present system captures scarce information from private healthcare facilities and, as a result, a significant portion of the true magnitude could be missing, thus further perpetuating the ‘tip of the iceberg’ phenomenon. Even when syndromes are reported, these are often underreported as many AGE cases tend to self-treat without presenting to a healthcare provider (13). The aetiology of the illness is also largely unknown in T&T as stool samples are almost never collected and tested for foodborne pathogens from patients presenting for care of AGE.

Accurate reporting is, therefore, necessary for advocating funds to implement prevention and control policies, monitor and evaluate current food safety measures, assess the cost effectiveness of the existing interventions, and quantify the burden in monetary costs.

The main objective of this study was to collect baseline data to determine the true community prevalence of AGE in T&T, to measure the burdens associated with this illness and quantify the level of underreporting. Other objectives included: assessing foodborne pathogens to determine the aetiology of the disease in T&T, which is largely unknown; evaluating the laboratory capacity, which enables timely and sensitive diagnosis of cases presenting with AGE, and identify gaps in the national surveillance system.

This was a joint study between University of the West Indies and the Ministry of Health in T&T. It was also part of the Caribbean Burden of Illness (BOI) study being coordinated by the Caribbean Epidemiology Centre and the Pan American Health Organization (CAREC/PAHO/WHO). Other collaborators were the International Development Research Centre (IDRC) and the Public Health Agency of Canada (PHAC).

## MATERIALS AND METHODS

The BOI study for AGE consisted of a retrospective cross-sectional population survey in T&T and a laboratory survey in 2009.

### Population survey

A cross-sectional population survey was conducted during the high-AGE season (15 November 2008–6 December 2008) and low-AGE season (15 May 2009–6 June 2009). The high- and low-AGE seasons were designated based on five-year trend data reported to the National Surveillance Unit (NSU) on weekly basis as the number of AGE cases in the community, who attended public hospitals and/or health centres. The population survey was administered to residents living in T&T by trained persons from the Central Statistical Office (CSO) by face-to-face interviews. A multistage sampling process was employed to select individuals to participate in the survey as follows: First, the country was divided into the government-designated five Regional Health Authorities (RHAs). Each RHA was then subdivided into Enumeration Districts (EDs) (geographical groupings of households—usually between 200 and 250 households); next, EDs were randomly selected with probability proportional-to-size in each RHA; within each ED, households were randomly selected in the field, using a random start and a constant interval. This was done by the Central Statistical Office (CSO) of T&T, that produced a listing of randomly-selected households by EDs.

In each household, the individual with the next birthday falling before the day of the interview was then selected to participate in the survey. If the chosen individual declined or did not respond after three attempts, the next immediate household was selected conveniently as a replacement. Individuals below the age of 12 years required the parents/guardians to answer on their behalf whereas individuals between the age of 12 and 18 years were permitted to answer at the discretion of the parents/guardians. All individuals over 18 years of age were allowed to answer for themselves. Persons unwilling or unable to participate, not physically present in the country at the time of survey, less than 18 years without parental consent, prisoners, and mentally-disabled persons, were excluded from the survey.

Sample-size was calculated using Epi Info (version 3.5.1) (Centers for Disease Control and Prevention, Atlanta, GA, USA). Using a population-size of approximately 1.3 million, prevalence for AGE of 50%, an expected response rate of 80%, allowable error of 3%, a 95% confidence interval, and a design effect of 1.5, the sample-size was calculated to be 2,145, which resulted in 1,067 individual surveys in the high season and 1,078 in the low season.

Data were collected by means of a standard questionnaire developed for the Caribbean BOI study. Respondents were asked if they had experienced any symptoms of AGE in the 4 weeks (28 days) prior to the interview date. AGE was defined as three or more loose stools in 24 hours with or without fever (14). Individuals who suffered from chronic diarrhoea or diarrhoea caused by the use of medications, laxatives, alcohol, or medical conditions were considered non-cases and excluded from analyses. Individuals were asked also about sociodemographic factors (age, sex, culture, education, and income), treatment and healthcare options, secondary symptoms experienced, days of restricted activity due to illness, hospitalization, illness status of other members of the household during the period, foods consumed, and food safety practices.

### Laboratory survey

A laboratory survey was administered via face-to-face interviews to the laboratory managers of the 6 major laboratories (Mount Hope Medical Sciences Complex, San-Fernando General Hospital, Port-of-Spain General Hospital, Tobago Regional Hospital, Sangre Grande Hospital, and the Trinidad Public Health Laboratory) that processed and tested more than 80% of all AGE cases and diarrhoeal stool samples throughout T&T. A standard questionnaire developed for the Caribbean BOI study, based on set questions used in previous laboratory surveys, was used for collecting information from the director of each laboratory selected. The survey obtained data on the number of diarrhoeal stool samples collected and tested; the proportion positive for AGE-causing pathogens and the proportion of those samples which were reported to the NSU for 2009.

During the study period, all AGE specimens tested in public laboratories were recorded by the lead investigator. However, to ensure that the study does not disrupt the normal operations of the surveillance system in T&T, all reporting to NSU was done via the public laboratories.

### Estimation of underreporting and the burden of AGE

**Figure 1.**
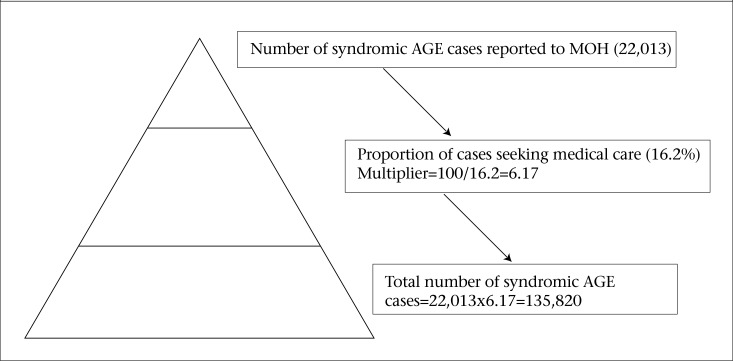
Burden and underreporting of syndromic AGE in the communities of Trinidad and Tobago

**Figure 2.**
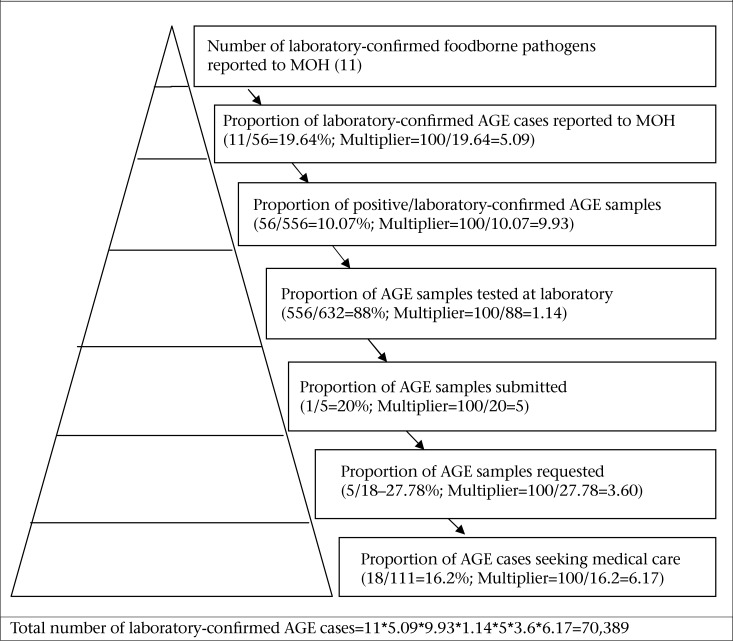
Burden and underreporting of laboratory-confirmed AGE pathogens in T&T

Syndromic AGE and laboratory-confirmed AGE data reported to the NSU as well as data from the population and laboratory surveys were used in calculating the burden and establishing the level of underreporting. From the population survey, the percentage of AGE cases who sought medical care (visited the public hospitals, health centres, and clinics) was employed to estimate the burden and degree of underreporting of syndromic AGE (Figure 1). The percentage of AGE cases who sought medical care, submitted a stool sample, stool tested at laboratory, stool positive for an AGE-related pathogen, and reported to the NSU were all used in estimating the underreporting for laboratory-confirmed AGE (Figure 2).

### Ethics

Ethical approval for the population survey was granted from the Ethical Review Board of University of the West Indies and Review Committee of the Ministry of Health. The data collected were kept confidential. The names of participants were not included on the questionnaire. Each participant was informed of the purpose of the survey and asked to sign a consent form before the questionnaire was administered.

### Statistical analyses

Data were manually entered into EpiData (version 3.1) (EpiData Association, Odense, Denmark) and Microsoft Excel (Microsoft Corporation, WA, USA) and managed using Epi Info (version 3.5.1). Individuals responding ‘don't know’ were excluded from analysis of that question. Demographic characteristics were compared with the 2000 population census data from the CSO (where available) to determine the representativeness of the study population with regard to the general population. Univariate analysis was performed on the dataset. Prevalence, incidence proportion and rate calculations were performed using standard formulae (15). Monthly prevalence was defined as: the number of participants reporting AGE in the previous 28 days/the total number of participants in the population survey. Prevalence and incidence rates were calculated using the formulae shown in Appendix 1. The null hypothesis of no association between prevalence of AGE and sociodemographic factors was tested using the chi-square test for statistical significance.

## RESULTS

### Response rate and representativeness of respondents

From the sample-size of 2,145 households selected to be interviewed, the overall response rate was 99.95%, with 1,067 individual surveys completed in Phase 1 (high season: 15 November–6 December 2008 and 1,077 in Phase 2 (low season: 15 May–6 June 2009). Fifty-one percent of the respondents were female, with 44.61%, 33.04%, and 19.04% being African, Indian, and mixed (African and Indian) descent respectively (data not shown). Comparison of the general population in T&T with the survey respondents indicate that the survey was overall well-represented (9). However, the age-category (25-44 years) was underrepresented (survey 28.0% : population 30.2%); females were overrepresented (survey 51.0% : population 50.1%); and males were underrepresented (survey 49.0% : population 49.9%). Approximately 24.0% of the respondents were from households earning a total monthly income of TT$ 5,000-7,500 while 8.0% and 18.2% of the respondents earned TT$ 2,500 and >TT$ 12,500 per month respectively. With regard to the education level of male and female household heads, most males (35.5%) and females (40.4%) attended high school (data not shown). More males (20.1%) compared to females (10.7%) had no formal education (data not shown). Data were not available from the Central Statistical Office to compare earnings and education levels of survey households with that of the general population.

### Magnitude and distribution of illness

Of the 2,144 respondents interviewed, 5.13% (n=110; 95% CI 4.3-6.2) reported that they had a sudden onset of diarrhoea (3 or more loose watery stools within 24 hours with or without fever, vomiting, or visible blood in stool) in the 4 weeks prior to the interview date. These persons were, therefore, classified as ‘self-reported’ cases of AGE. The annual incidence rate was 0.6748 episodes per person-year, with 0.7083 episodes per person-year in males and 0.6321 episodes per person-year in females. Self-reported AGE cases were asked to identify what they believed to be the cause of their illness. The major reasons reported were: something they consumed (35.1%), drinking-water (17.1%), contact with another sick person (9.9%), and contact with an animal (9.9%) (data not shown). Only one person believed the illness was due to a bacterial infection.

The monthly prevalence of AGE by demographic factor is outlined in Table 1. A univariate analysis was conducted on each of the sociodemographic factors to test its effect on AGE. Relationships were considered significant at p<0.05. The study revealed that only age-group had a significant effect on AGE. The highest monthly AGE prevalence of 13.3% was observed in cases aged <1 year, and the lowest (3.6%) seen in cases aged 45 years and older (Table 1). As a result of over- and underrepresentations found in the study, the prevalence was weighted to represent the entire population. Hence, the prevalence adjusted for age and sex was 5.68% and 5.1% respectively (Table 2 and 3). No significant differences were found between the adjusted prevalence and prevalence for age and sex found in this study.

### Symptoms and severity

The most commonly-reported duration of diarrhoea (66%) was three days, with a range of 1-10 day(s). Abdominal pain was the most common secondary symptom (64.9%), followed by vomiting (29.7%), headache (26.1%), and fever (25.2%). Respiratory symptoms were less common (7.2-20.7%), and blood in stool was somewhat uncommon, affecting 6.3% of respondents with diarrhoea (Table 4). Approximately 66% of cases reported restricted activity, and, on average, restriction of normal activity lasted ~3 days, with a minimum of 1 and a maximum of 16 days (Table 2). Time spent away from normal activities due to this illness can result in: medication and medical visit costs, costs for a caretaker, loss of leisure activity, loss of income for the working population, and loss of days from school. All of these factors can pose serious social and economic impacts on the individual and the country as a whole. Twenty-six cases required other individuals to look after them while ill, and the range for taking care of a case was 1-8 day(s), averaging 2.6 days. A total of 1.47% other household members of respondents also reported to have experienced AGE within the past 4 weeks (data not shown).

### Healthcare-seeking behaviour/Use of medical systems

Approximately 18 (16%) of cases sought medical care for their illness. One attended a private hospital, 5 attended public hospitals, 8 attended private doctors’ clinics, one attended a health centre, and 3 went to the pharmacies (data not shown). None visited a traditional healer or an alternative healthcare practitioner. One case was hospitalized for 4 days. Seventeen of the 18 cases had medication prescribed to them. Six were prescribed antibiotics (37%), two of whom completed the course of antibiotics and four of whom did not. Four individuals (25%) were prescribed oral rehydration salts (ORS) while another four were prescribed pain killers. Cases not seeking medical care for their illness took non-prescribed medications, such as pain killers (25%), ORS (17%), ‘unknown bush medicine’ (8%), Lomotil (9%); Imodium (8%), Ridol (2%), and 4% drank a combination of flour and water (data not shown). The WHO recommends that ORS should be the effective method chosen to deal with AGE as diarrhoea is self-limiting and will usually go away after 3 days.

**Table 1.** Demographic characteristics of residents and survey respondents, and monthly prevalence of acute self-reported gastrointestinal illness by category in T&T

Variable (n) (p value)	Residents N (%)	Survey respondents (%)	(%) Monthly prevalence of AGE (n)	95% Confidence interval
Sex (n=2,142) (p=0.3032)	1,114,772 (100)	2,142 (100)	5.2 (110)	4.3-6.2
Male	556,110 (49.9)	1,049 (49.0)	5.4 (57)	4.2-7.0
Female	558,662 (50.1)	1,093 (51.0)	4.8 (53)	3.7-6.3
Age (completed years) (n=2,139) (p=0.0335)				
<1	**	15 (0.7)	13.3	1.7-40.5
1-4	76,508 (6.9)	90 (4.2)	9.5 (10)	4.7-16.8
5-14	207,738 (18.6)	204 (9.5)	8.3 (17)	4.9-13.0
15-24	221,649 (19.9)	290 (13.6)	5.5 (16)	3.2-8.8
25-44	336,542 (30.2)	599 (28.0)	5.5 (33)	3.9-7.7
45-64	194,691 (17.4)	633 (29.6)	3.6 (23)	2.4-5.5
≥65	77,644 (7.0)	308 (14.4)	3.6 (11)	1.9-6.5
Cultural group (n=2,135) (p=0.9075)				
African/Black decent	418,268 (37.5)	956 (44.8)	5.1 (49)	3.9-6.8
Indian decent	446,273 (40.0)	708 (33.2)	5.4 (38)	3.9-7.4
Asian decent	3,800 (0.34)	18 (0.8)	5.6 (1)	0.1-27.3
Mixed (African/Indian) decent	228,089 (20.5)	408 (20.1)	4.7 (19)	2.9-7.3
South American decent	NA	8 (0.4)	12.5 (1)	0.3-52.7
North American decent	7,034 (0.63)	28 (1.3)	7.1 (6)	0.9 −23.5
Other	*1,972 (0.18)	9 (0.4)	0 (0)	-
Income US$ (n=1, 894) (p=0.1150)				
Low (0-385)	**	151 (8.0)	4.6 (7)	1.9-9.3
Low-medium (386-770)	**	418 (22.1)	3.8 (16)	2.3-6.3
Medium (771-1,154)	**	453 (23.9)	6.0 (27)	4.0-8.7
Medium-high (1,155-1,538)	**	328 (17.3)	5.5 (18)	3.4-8.7
High (1,539-1,923)	**	199 (10.5)	2.0 (4)	0.6-5.1
Very high (>1,924)	**	345 (18.2)	7.5 (26)	5.1-11.0
Education (Mother) (n=1,844) (p=0.1320)				
Primary	**	683 (37)	5.7 (39)	4.1-7.8
Secondary	**	836 (45)	5.0 (42)	3.7-6.8
Certificate/Diploma	**	179 (9.7)	6.7 (12)	3.5-11.4
Undergraduate/Graduate	**	82 (4.4)	7.3 (6)	2.7-15.2
Postgraduate	**	64 (3.5)	1.6 (1)	0.0-8.4
Education (Father) (n=1,623) (p=0.1581)	**			
Primary	**	584 (36.0)	6.3 (37)	4.6-8.7
Secondary	**	722 (44.5)	5.0 (36)	3.6-6.9
Certificate/Diploma	**	182 (11.2)	2.7 (5)	0.9-6.3
Undergraduate/Graduate	**	66 (4.1)	10.6 (7)	4.4-20.6
Postgraduate	**	69 (4.3)	1.4 (1)	0.0-7.8
Mean number of people living in household	3.65	3.4		
Mean number of other people in house with diarrhoea	**	2.6		

Data were not always given by interviewer during the interview (for example, age, income, etc.) and, therefore, the total will not always add up to 2,144;

*6.7% [value given by the CSO for the age-group 0-4 year(s)];

*Population data not available from Central Statistical Office;

NA=Not available

**Table 2.** Age-group-adjusted rate of monthly AGE prevalence in Trinidad and Tobago

Age-group (in completed years)	Population (%)	Rate (%)	Age-group-adjusted monthly prevalence (Rate/100)
0-4	0.067	9.5	0.637
5-14	0.152	8.3	1.262
15-24	0.196	5.5	1.078
25-44	0.314	5.5	1.727
45-64	0.235	3.6	0.846
≥65	0.036	3.6	0.130

The age-group-adjusted rate of monthly AGE prevalence in Trinidad and Tobago is 5.68%

**Table 3.** Sex-adjusted rate of monthly AGE prevalence in Trinidad and Tobago

Sex	Population (%)	Rate (%)	Sex-adjusted monthly prevalence (Rate/100)
Male	0.502	5.4	2.71
Female	0.498	4.8	2.39

The sex-adjusted rate of monthly AGE prevalence in Trinidad and Tobago is 5.10%

**Table 4.** Secondary symptoms, duration, and severity of AGE symptoms in T&T

Secondary symptoms (n=111)	Number of cases (%)
Fever (measured)	8 (7.2)
Fever (not measured)	28 (25.2)
Blood in stool	7 (6.3)
Vomiting	33 (29.7)
Abdominal pain	72 (64.9)
Headache	29 (26.1)
Nausea	22 (19.8)
Sore throat	8 (7.2)
Cough	23 (20.7)
Runny nose	20 (18.0)
Sneezing	17 (15.3)
Duration of diarrhoea	Days
Mean	3
Median	4
Range	1-10
Length of restricted activity due to diarrhoea	Days
Mean of restriction to home	2.9
Median of restriction to home	5.5
Range of restriction to home (days)	1-16

### Underreporting estimation and overall burden of AGE

Table 5 summarizes results of the epidemiology of AGE found in this study, using the standard case definition of symptomatic AGE. Assuming that all cases who sought medical care were reported to the NSU, the estimated number of AGE cases in the population for 2009 was 135,820 (Figure 1). Given that the reported number of cases in 2009 was 22,013, this estimate implied that, for every one case of AGE reported to the NSU, an additional 6.17 cases occurred in the community. Hence, the percentage of cases not reported is 83.79. When extrapolated to the population, approximately 10% (one in every 10 persons) gets AGE each year in T&T.

**Table 5.** Epidemiology of syndromic AGE, using the standard case definition, T&T, 2009

Category of minimum set of results	Value
Annual incidence/person-year	0.6748
Annual incidence/person-year in males	0.7083
Annual incidence/person-year in females	0.6321
Mean age of cases (years)	0-4
Mean duration of illness (days)	2.3
Cases with bloody diarrhoea (%)	7.1
Cases who saw a physician (%)	16.2
Cases who submitted a sample for testing (%)	20
Number of self-reported cases of AGE	110

The number of confirmed AGE-related pathogens reported to the NSU in T&T during the period January 2009 to December 2009 was 11. However, 56 (10%) of AGE specimens tested during this study were positive for a foodborne pathogen. This finding indicates a gap in reporting to NSU. Therefore, the ‘true’ number of laboratory-confirmed cases of AGE occurring in the communities of Trinidad and Tobago was estimated to be 70,389, with an underreporting factor of 6,399 (Figure 2). This estimate implied that foodborne pathogens found in this study accounted for approximately half of all AGE cases in Trinidad and Tobago for 2009. Foodborne pathogens found included: *Salmonella, Shigella,* rotavirus, and norovirus.

### Economic impact of AGE in Trinidad and Tobago

The estimated economic burden of AGE was based on AGE patients visiting a private healthcare facility. Although public healthcare facilities provide free services to the public, there is still a cost to the state for medical services and supplies. The calculations were done taking into consideration the minimum expenses required for basic private medical care (Appendix 2). The estimated cost for one case ranged from US$ 1 to 155. Therefore, the estimated economic burden for AGE was US$ 135,820-21,052,100 for 2009.

## DISCUSSION

This study provided the first population-based estimates for the magnitude, distribution, and burden of acute gastroenteritis (AGE) in the communities of Trinidad and Tobago (T&T). The findings provide empirical evidence that AGE is a significant public-health issue and can be an economic burden on the state. It also highlighted major gaps in surveillance, such as untimely and incomplete reporting to the NSU as well as the infrequent/non-existent request and collection of stool samples by doctors from AGE cases. In addition, many persons seeking private healthcare for AGE are not captured by the NSU, thus further resulting in the underreporting of the disease.

From literature review (16-24), there were many different study designs and case definitions used in determining the burden of self-reported AGE. However, these case definitions were comparable to other retrospective, population-based Burden of Illness (BOI) surveys conducted in Chile, Cuba, Canada, Australia, Ireland, Norway, USA, and Jordan. The AGE prevalence of 5.2% and incidence of 0.6748 episodes per person-year reported in T&T were approximately less than half of the overall AGE prevalence and incidence rates occurring in other countries. Studies done in Cuba, Chile, and Canada had AGE prevalence of 10.6%, 9.2%, and 9.2% respectively while studies in Norway, Canada, and USA found AGE annual incidence rates of 1.2, 1.3, and 1.4 episodes per person-year respectively. A study done by Scallan *et al*. (19) in Ireland, however, reported an incidence rate of 0.6 episodes per person-year, which was similar to that found in this study.

The estimated burden of syndromic AGE and laboratory-confirmed AGE pathogens were 135,820 and 70,389 respectively. These figures were substantially higher than that reported to the NSU (22,013 and 11 for AGE and AGE-related pathogens respectively). Using the minimum expenses required for one case seeking basic private medical services (US$ 1-155), the estimated burden of syndromic AGE for 2009 in T&T was US$ 135,820-21,052,100.

Although public healthcare facilities provide free services to the residents of T&T, there is still a monetary burden to the state, such as for maintenance of health facilities and purchase of drugs and supplies. AGE ranks second in the list of communicable diseases, fifth in morbidity profile in T&T (25) and, according to this study, affects approximately 10% of the population each year, resulting in millions of dollars for treatment. In Canada (26) and the Netherlands (27), this costs approximately the same amount to treat one case of AGE (US$ 110 and 106 respectively). In the United States of America (28), costs associated with one bacterium (*Clostridium difficile*, which is related to nosocomial diarrhoea), exceed 1.1 billion/year whereas, in Australia (29), visits to the general practitioners for AGE costs US$ 26,722,691 each year. Although expenses associated with AGE may be lower than that for other diseases, such as malaria (direct costs are at least US$ 12 billion per year) (30) and tuberculosis (costs to the European Union are at a conservative US$ 7.96 billion per year) (31), it does represent a substantial burden considering that it is a preventable disease.

Understanding the relationship between AGE and demographic determinants is necessary to guide prevention and intervention efforts as considerable costs and significant public-health and economic burdens are borne. Greater efforts should be made to improve the AGE surveillance system in T&T so that timely and accurate information is provided for detecting and preventing AGE in T&T.

Our estimates implied that foodborne pathogens accounted for approximately half of AGE cases in T&T. In 2010, T&T's bill for food import amounted to approximately 10% of the total import (32). The problem arises when little is known about the place of manufacture where substandard sanitary conditions could exist. In addition, people are busier and have less time to prepare meals, perhaps leading to eating out more frequently than in the past, thereby increasing the chance of a person being susceptible to the bad habits of careless food-handlers (33).

The occurrence of AGE outbreaks would provide more information on the aetiology and geographic distribution of AGE-related illness in T&T. Outbreaks can indicate the pathogens and foods that most likely caused morbidity, hospitalizations, and deaths. Trends over time can also be predicted. The pathogens isolated in this study are also commonly found during outbreaks and can be directly linked to foods as well as hygiene practices. Salmonellosis and shigellosis are infections caused by bacteria called *Salmonella* and *Shigella* respectively and have been known to cause illness for many decades. Most of these two infections can be spread by person-to-person contact via the oral-faecal route. There are no vaccines for both bacteria; however, frequent handwashing with soap may stop the spread. Some doctors may even prescribe a course of antibiotic to treat the infection. Cross-contamination of foods should be totally avoided. Uncooked meats should be kept separate from produce, cooked foods, and ready-to-eat foods. Foods of animal origin may be contaminated with *Salmonella*, and people should avoid eating raw or undercooked eggs, poultry, or meat.

Rotavirus and norovirus were also isolated in this study. Rotavirus is the leading cause of severe diarrhoea in infants and young children worldwide. It causes severe watery diarrhoea, often with vomiting, fever, and abdominal pain. In babies and young children, it can lead to dehydration (loss of body-fluids). While handwashing and cleanliness are important ways to stop the spread of germs, this is not enough to stop rotavirus. The best way to protect, especially children, is to get vaccinated. Currently, the vaccine is not available in T&T. Norovirus is also very contagious and can spread from an infected person, contaminated food or water, or for touching contaminated surfaces. Moreover, proper hygiene is needed to prevent its spread. Fruits and vegetables should be thoroughly washed and cooked properly before consumption.

Based on the symptoms presented by the self-reported AGE cases, it was difficult to determine the particular pathogens that caused their illness as many pathogens may cause common symptoms of nausea, vomiting, diarrhoea, abdominal cramps, etc. Only through laboratory testing can pathogens be confirmed.

Our study revealed that age-group was significantly associated with AGE while other sociodemographic factors were not. The highest monthly AGE prevalence of 13.3% was observed in cases aged <1 year and the lowest prevalence of 3.6% seen in cases aged 45 years and older. This finding was consistent with international trends where a higher AGE prevalence was usually observed among young children (4,16-20,34,35) and reflects an increased susceptibility due to immunological naivety (36). In teenagers, however, the risk could be more associated with behavioural factors, such as poor hygienic practices that result in ingestion of contaminated food and water (37). The tendency to be exposed is greater for infants and young children who are cared for by providers with poor hygiene habits or cross-exposure to other children in day-care centres and schools (36). Smith (38) revealed that a lower AGE prevalence among the elderly can be partly due to older persons being more careful about food-handling and food consumption than younger ones.

The symptoms, severity, and duration of AGE in T&T were similar to studies previously done (16-20,34,38). Sometimes, AGE symptoms can give an inclination of the pathogen(s) most likely to having caused illness. To confirm the causative agent, laboratory testing must be done and, in many cases, this is done via stool testing. Although persons may experience diarrhoea, it is frequently unvoiced (39) and, as a result, persons are reluctant to discuss their diarrhoeal episodes and/or submit a stool sample for testing. These factors can, therefore, result in a gap of aetiological data. The observed duration of illness in this study was lower than that reported in Canada (17) but similar to a study done in Chile (16).

The percentage of those not seeking healthcare and submitting a stool sample was similar to another study where ‘the illness was not important enough’ as it could be treated at home (37). Self-treatment was higher in this study than studies reported in Cuba and Canada. Time spent away from normal activities due to this illness can result in considerable costs, including medication and medical visit costs, costs for a caretaker, loss of leisure activity, loss of income for the working population, and loss of days from school. These factors can pose serious social and economic impacts on the individual and the country as a whole.

This study also highlighted several gaps in T&T surveillance system. These included: untimely and incomplete reporting of laboratory data from the hospitals to NSU, non-compliance by doctors in requesting stool samples from patients as well as patients not submitting stool samples. Doctors do not commonly request a stool sample, and patients were also reluctant to submit samples. Laboratory capacity to process the samples was also insufficient. There was difficulty in obtaining records from the hospital's laboratory as no computerized system exists, and doctors also frequently neglect to complete the laboratory reporting forms. In addition to the small number of samples being submitted, this study observed that approximately 4% of stools were rejected due to inappropriate labelling and storage during transportation. These factors could negatively affect the integrity of the stool samples since pathogens that may be present could by then be deteriorated or dead (40). Administrative roadblocks also led to delayed reporting from the Trinidad Public Health reference laboratory to the NSU.

### Limitations

A potential shortcoming of this study was the retrospective methodology employed. Research (41) indicates that the retrospective methodology may be subjected to more bias than the prospective method and, as such, the prospective methodology is preferred. However, this is compensated for by the fact that this method has been used with success in numerous other retrospective Burden of Illness (BOI) studies (4,16-21,35), thereby enabling comparisons with these studies to establish global AGE estimates.

Another potential limitation was in the sampling method used. The sampling was done with replacement, which allowed a household selected in the sample to be placed back in the population to be possibly sampled again. However, no single household was selected twice during the interview process.

### Conclusions

AGE pose significant economic and health burden on T&T. To reduce the burden and morbidity associated with AGE in T&T, the following recommendations should be considered: educational campaigns targeting both doctors and patients to improve specimen collection; hygiene interventions that target the general public; and doctors properly filling out laboratory forms. Risk factors of AGE should be identified and resources distributed appropriately. To streamline the current surveillance system, samples should be collected in sterile containers, stored at appropriate temperatures, and processed within acceptable time limits to detect pathogens that may be present. Additionally, results from the reference laboratories should be sent to the NSU on time to facilitate the continuous systematic collection, analysis, and interpretation of health-related data needed for the planning, implementation, and evaluation of public-health practice.

These data can serve as an early warning system for impending public-health emergencies and can be useful in monitoring and clarifying the epidemiology of health problems to allow priorities to be set and to inform public-health policy and strategies (42).

Finally, the information gained from this study should be disseminated among colleagues in the public health sector (possibly to prevent similar outbreaks in future) and among all people along farm-to-fork continuum through public awareness campaigns.

## ACKNOWLEDGEMENTS

The authors would like to express gratitude to CAREC/PAHO for providing technical guidance to this study. We deeply acknowledge the Caribbean Eco-Health Programme, the IDRC's Teasdale Corti grant, University of the West Indies, and CAREC/PAHO for funding this study. Thanks are also due to the following collaborating institutions for the successful completion of the study: Ministry of Health, Central Statistical Office, Trinidad Public Health Laboratory, laboratories of the public hospitals, and the Public Health Agency, Canada. Special thanks to Ms Meagan Hatcher from the University of Guelph for assisting in the editing of this paper.

## Figures and Tables

**Appendix 1.**
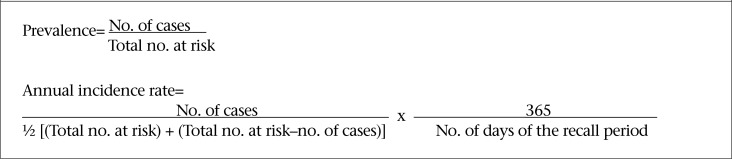
Formulae for calculating prevalence, incidence rate, and incidence proportion

## Appendix

**Appendix 2.** The estimated economic burden of AGE was based on AGE patients visiting a private healthcare facility. The calculations were done taking into consideration the minimum expenses required for basic medical services and supplies. The estimated cost of one case at private healthcare ranged from US$ 1 to 155

Activities by AGE patients	Approximate cost (TT$)
Medical visit	120-500
Medication cost (overall)	430
ORS	10
Antibiotic	40
Gravol	6 (200-injection form)
IV	85/bag
Pain killer	9 (200-injection form)
Stool test (overall)	280
Culture	150
Parasites	70
Blood	60
Lost work (based on average three days lost income)	
Low	375/3 days/AGE case
Middle	875/3 days/AGE
High	1,375/3 days/AGE
Caregiver	80/day/AGE case

Public healthcare facilities provide free services to the public; however, there is still a cost to the state; Rates calculated based on estimates from population survey.
Number of AGE-affected persons who visited public facilities=6/18=33.3% Number of persons who visited private facilities=9/18=50% Income categories of persons: ▪ Low income=569/1,894=30.1% ▪ Middle income=781/1,894=41.2% ▪ High income=544/1,894=28.7% Caregiver=23.4% @ US$ 10 (minimum wage for 8 hours a day/case)
